# Memories of Terry Erwin

**DOI:** 10.3897/zookeys.1044.68648

**Published:** 2021-06-16

**Authors:** John Spence, David Kavanaugh, David R. Maddison, Olivia Boyd, Pietro Brandmayr, Beulah Garner, Sarah Maveety, Diego Mosquera, Wendy Moore, Kathryn Riley, Joseph Shorthouse, Linda Sims, Warren Steiner, Kelly Swing, Hans Turin, Laura S. Zamorano, Lyubomir Penev

**Affiliations:** 1 University of Alberta, Edmonton, Canada; 2 Californian Academy of Sciences, San Francisco, United States of America; 3 Oregon State University, Corvallis, United States of America; 4 Oregon State University, Corvallis, United States of America; 5 Università della Calabria, Rende, Italy; 6 Natural History Museum, London, United Kingdom; 7 Brevard College, Brevard, North Carolina; 8 Universidad San Francisco de Quito, Quito, Ecuador; 9 University of Arizona, Tucson, United States of America; 10 Pfeiffer University, Misenheimer,United States of America; 11 Laurentian University, Sudbury, Canada; 12 Smithsonian Institution, Washington, United States of America; 13 Universidad San Francisco de Quito, Quito, Ecuador; 14 Unaffiliated, Renkum, Netherlands; 15 Centre national de la recherche scientifique, Paris, France; 16 Institute of Biodiversity & Ecosystem Research – Bulgarian Academy of Sciences, Sofia, Bulgaria; 17 Pensoft Publishers, Sofia, Bulgaria

## Abstract

We were fortunate to have known Terry not only as an excellent professional coleopterist and an enthusiastic colleague, but also as a good friend. Entomological meetings for us came with an evening supper or two with Terry and the kind of laid-back personal catch-up that happens only among friends with long-term interest in each other’s lives. Through our connections with the University of Alberta and George Ball we were also happy members of Terry’s basal academic family. While we will join the rest of a broader scientific community in missing his presence in development of ideas about beetles, biodiversity and evolution, the kinds of work that Terry promoted will continue. We will, of course, be interested in following how the understanding of carabids and nature develops further from Terry’s contributions. This will most certainly continue to grow, partly through the efforts of those that he has influenced. Every practicing research scientist has some role to play in the great chain of discovery, and much of this volume is meant to celebrate Terry’s contributions and showcase how they have influenced the work of others.

Our own more enduring sense of loss will flow from the personal interactions with Terry that were generally part of our timelines. Despite the sadness associated with such loss, our memories of interactions with Terry underscore a sense of joy and gratefulness for having connected with him interpersonally in life. Given Terry’s affable and social nature, many others will have such memories. Thus, when Lyubomir Penev asked us to coordinate a selection of ‘memories’ for this memorial volume, we were happy to undertake the task and gather together a selection of memories of our friend, Terry Erwin. What follows is a series of recollections by people who knew and worked with him from a number of perspectives during a broad range of his academic career.

We are most grateful to those who have been willing to share their reflections. These are presented here as a way of reaching beyond Terry’s considerable scientific influence to also preserve some sense of his influence on the lives of people, and the ways in which he encouraged and inspired them. We thank all the contributors for their efforts and Diane Hollingdale for work to bring the included photographs to the best possible publication standard.

John R. Spence

Edmonton, Alberta

David H. Kavanaugh

San Francisco, California

David R. Maddison

Corvallis, Oregon

## Introduction

We were fortunate to have known Terry, not only as an excellent professional coleopterist and an enthusiastic colleague, but also as a good friend. Entomological meetings for us came with an evening supper or two with Terry and the kind of laid-back personal catch-up that happens only among friends with long-term interest in each other’s lives. Through our connections with the University of Alberta and George Ball we were also happy members of Terry’s basal academic family. While we will join the rest of a broader scientific community in missing his presence in development of ideas about beetles, biodiversity and evolution, the kinds of work that Terry promoted will continue. We will, of course, be interested in following how the understanding of carabids and nature develops further from Terry’s contributions. This will most certainly continue to grow, partly through the efforts of those that he has influenced. Every practicing research scientist has some role to play in the great chain of discovery, and much of this volume is meant to celebrate Terry’s contributions and showcase how they have influenced the work of others.

Our own more enduring sense of loss will flow from the personal interactions with Terry that were generally part of our timelines. Despite the sadness associated with such loss, our memories of interactions with Terry underscore a sense of joy and gratefulness for having connected with him interpersonally in life. Given Terry’s affable and social nature, many others will have such memories. Thus, when Lyubomir Penev asked us to coordinate a selection of ‘memories’ for this memorial volume, we were happy to undertake the task and gather together a selection of memories of our friend, Terry Erwin. What follows is a series of recollections by people who knew and worked with him from a number of perspectives during a broad range of his academic career.

We are most grateful to those who have been willing to share their reflections. These are presented here as a way of reaching beyond Terry’s considerable scientific influence to also preserve some sense of his influence on the lives of people, and the ways in which he encouraged and inspired them. We thank all the contributors for their efforts and Diane Hollingdale for work to bring the included photographs to the best possible publication standard.

**John R. Spence**

Edmonton, Alberta

**David H. Kavanaugh**

San Francisco, California

**David R. Maddison**

Corvallis, Oregon

## “You be Bates; I’ll be Wallace!”

In the summer of 2017, at the edge of the Amazon Basin, I had the privilege of getting to know Terry Erwin over the course of a month of beetle collecting and conversations about fractal universes, the history of carabidology, and bird trivia.

Tiputini Biological Station, occupying ca. 600 hectares of lowland rainforest on the border of Ecuador’s Yasuní National Park, is the most remote place I have ever been. Terry seemed completely at home there. His knowledge of this area, gathered from several decades of research about every layer of the forest, was more of a friendship. He not only knew exactly where ’The *Asklepia* Spot‘ was without consulting GPS but could relate in detail how that particular microhabitat had shifted phenologically, hydrologically, and with respect to human activity over the years. This proved to be the case for, well, every ‘beetley’ spot along the many miles of rugged trails at Tiputini. He made a point to visit each of these spots and check in on their inhabitants every time he made the trip down. To everything he did, Terry brought a sense of humor, playfulness, and imagination, but also a deep interest in the history of tropical exploration and reverence toward the natural world. One afternoon, as we paddled out of the blackwater swamp back into the sunshine after a muddy six-hour pursuit of *Eucamaragnathus* and a close encounter with a rufescent tiger heron, Terry leaned back in the canoe and mused on what 19^th^ century entomologists like Wallace and Bates might have thought of the dream scene of kapok trees, bromeliads, and butterflies before us.

It wasn’t until one of our last days that I realized that the group I had somewhat arbitrarily chosen to study was exactly the right one. After extracting myself out of knee-deep mud onto something closer to terra firma, I finally struck gold and was able to not only collect a large series of the undescribed species I had been looking for, but to watch them for a while in their ephemeral habitat. Data that can only be gathered on one’s belly beside a collapsing riverbank are as much a part of what characterizes a species as the observations and measurements taken of a dead specimen under a microscope. The manic joy of fieldwork was what got me into entomology, but it was not until then that I really felt I understood its intellectual value. I later began to see the common thread running through Terry’s published work and that of his academic predecessors and their other students. Just as Darlington (1971, Systematic Biology 20: 341–365) described of Lindroth, Terry treated “his dead, dry specimens as representing real, living species, each with its own specific requirements, history, behavior, and significance.”

A month with Terry showed me the kind of teacher I want to be. His improvisational, casually Socratic style, interspersed with anecdotes about his own successes and failures and tales from his expeditions with George Ball, disarmed me into absorbing more information than I ever had in any college or graduate course. When I resumed teaching later that summer with this in mind, I found myself far less preoccupied with knowing all the answers and far more oriented toward pointing out compelling questions.

I think my life has largely been defined by the great naturalists I have known, and I have not really thanked most of them. Too often, it isn’t until we lose friends and mentors that we take a full inventory of all they’ve given us. I can only hope that Terry could read the gratitude on my face when I returned to the lab with that vial full of tiny spotted *Tachyura*. As great a naturalist and as prolific a taxonomist as he was, the way he honored his forebearers and gushed about his students told me that people were way more important to Terry than the beetles were. That is a lot of love for one person to put out into the world.

Thanks for everything, Terry!

***Olivia Boyd*** *is a Ph.D. candidate in David Maddison’s lab at Oregon State University in Corvallis, Oregon. Terry Erwin taught her to appreciate the personalities of neotropical tachyine carabids in 2015, co-authored her very first paper in 2016, and kindly withheld criticism of her canoe paddling ’skills‘ in 2017.*

**Figure:**
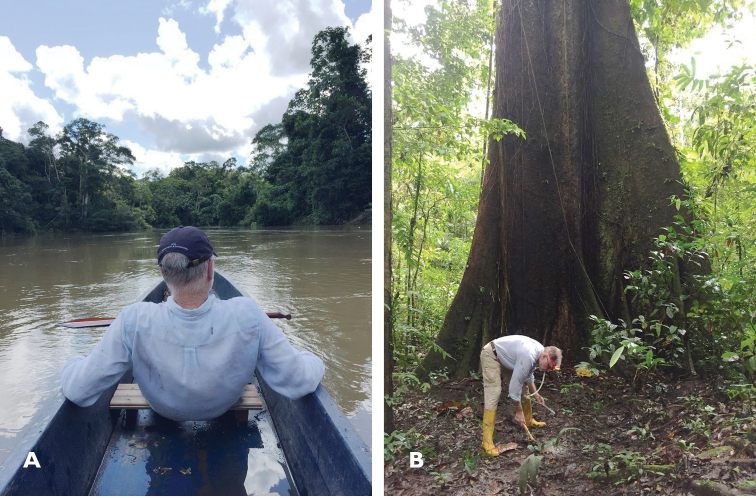
**Photograph captions. A** Floating back to the Estación de Biodiversidad Tiputini, Orellana, Ecuador on the Rio Tiputini after a morning of collecting in the blackwater swamp upstream of the station, 30 June 2017 **B** Terry Erwin aspirating small carabids (*Meotachys* and *Oxydrepanus*) from leaf litter, Sendero Chichico, 21 June 2017. Photographs: O. Boyd.

**Figure:**
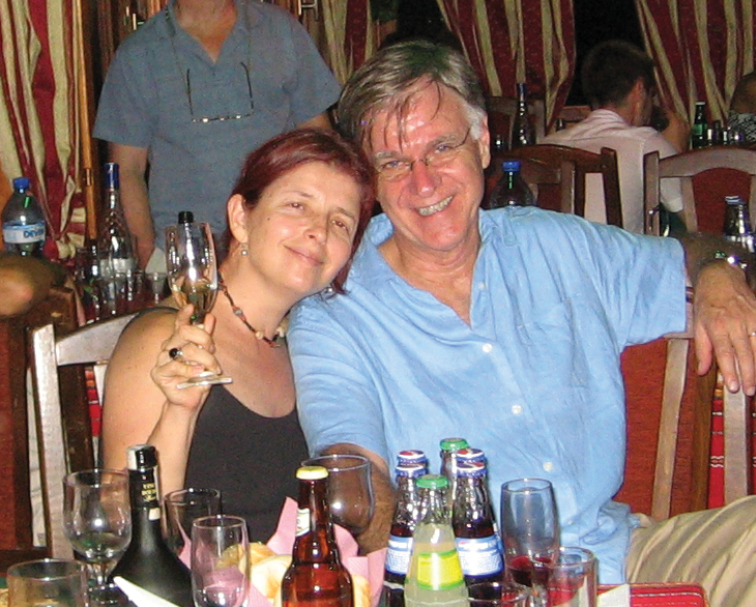
**Photograph caption**: Grace Servat and Terry Erwin at the social dinner of the XIII^th^ European Carabidologists meeting in Blagoevgrad, Bulgaria. August 2007. Photograph: J. Spence.

## Remembering Terry Erwin, an unforgettable carabidologist

I first encountered Terry during the XVII^th^ International Congress of Entomology in Hamburg, in 1984. Terry was for me a sort of unattainable example of what a carabidologist could be, a famous explorer of tropical wilderness and its still mysterious biodiversity. Only later, especially during the XIII^th^ European Carabidologists Meeting, held in Blagoevgrad (Bulgaria), I had the occasion to speak a little more with him during some evenings after having had dinner together with him and his gentle wife Grace Savant (see photograph). At this time he was focused on the enigmatic Pseudomorphini and describing several new genera of this poorly known tribe. I had questions about the original habitat of carabids and their evolutionary pathways, and we discussed a lot about ’taxon pulses‘ in the tropics and the differences in patterns seen in the temperate world. I very much enjoyed his passionate fondness for such topics and our enthusiastic discussions of anything, and what seemed to be ‘everything’, that could explain the way of life of the beetles of the megadiverse Carabidae.

Terry was always forward looking when speaking on such topics, and his prodigious scientific productivity bears witness to his yearning for new knowledge about systematics, phylogeny, and lifestyles of poorly investigated taxa. I remember especially some masterpieces, like the hunting behavior of *Cicindis* (fairy shrimp hunting beetle) and its ability to skate on the water (Zootaxa 553: 1–16), or his capacity to stimulate worldwide studies on ground beetle bionomics, as in Mawdsley et al.’s study of (2011) the natural history of the ’peaceful giant ground beetle‘: *Tefflus meyerlei* (Insecta Mundi 0181: 1–7). However, his most beloved issue was perhaps carabid life in tropical canopies and, in particular, the lebiine genus *Agra*, that presently numbers more than 600 described species but includes more than 1500 species presently undescribed. In his last scientific talk in Europe, at the XIX^th^ European Carabidologists Meeting in the Dolomites (2019, Fiera di Primiero), he told us many new things about the extreme nature of the canopy environment, sharing with us the deeper understanding that had resulted from his most recent work.

Dear Terry, carabidology owes you a lot. I am sure that you are seeking carabids in the canopies of celestial forests, to satisfy your thirst for knowledge about the evolution of carabids. In his book about the afterlife, Eben Alexander (2012, “Proof of Heaven: A Neurosurgeon’s Near-Death Experience and Journey into the Afterlife”) writes only of “millions of butterflies”, but in his creative fantasy the Lord and master of biodiversity certainly provided for us some billions of beetles! Have a good time!

***Pietro Brandmayr*** *is a retired professor from the Università della Calabria in Rende, Italy who was a long-time carabidological colleague and friend of Terry Erwin.*

**Figure:**
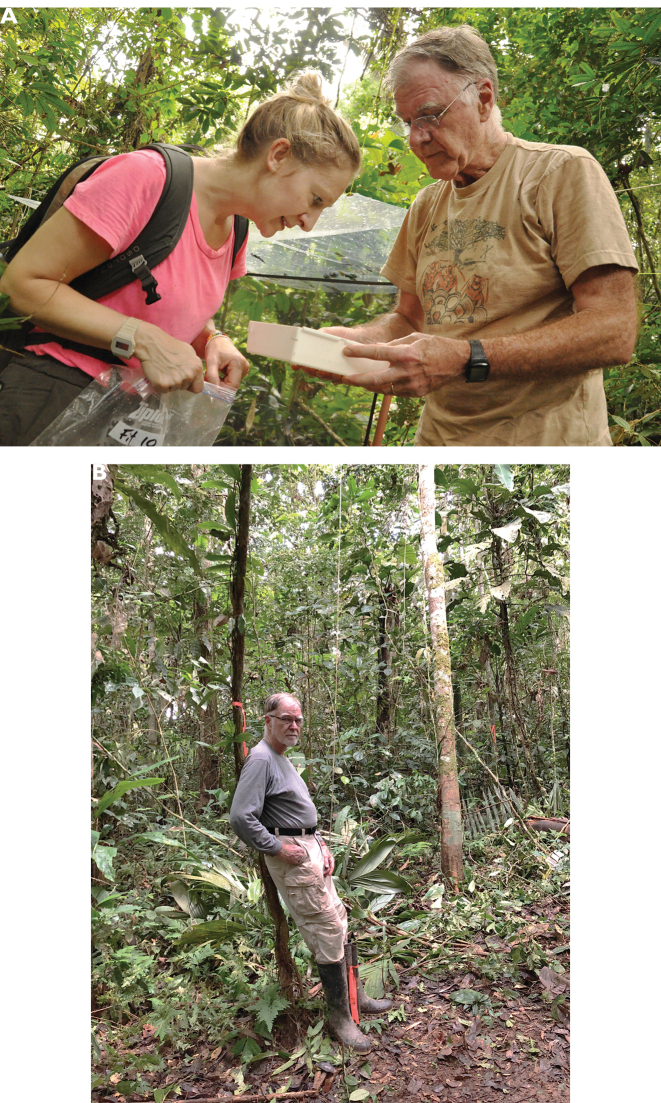
**Photograph captions.** Memories from the Tiputini Biodiversity Station in the Yasuni Biosphere reserve in eastern Ecuador **A** Terry Erwin and Beulah Garner viewing the captures from a flight intercept trap (FIT) set in a terra firme plot, 27 November 2013. Photograph: K. Swing **B** Terry in the Onkane Gare plot in Yasuni, 2018. Photograph: B. Garner.

## My Indiana Jones of Carabidology

It was my great privilege to have had the opportunity to have learnt from Terry. I was taken under his wing like so many students before me. He had the greatest spirit of academic generosity and was quick to provide advice. Whether it was a reference from his encyclopedic library or specimens for one’s own research. He was always contactable and entirely approachable. A rare beast given his status as a leading expert in his field.

Terry was a creature of routine, both in and out of the field. It was this routine, and of course his passion for entomology and forest ecology that helped him to produce such a huge body of work. In the field, Terry would wake with the dawn, and sit by the Tiputini River of Ecuador, with black coffee and binoculars in hand, and study the jungle whilst it awoke. He was indefatigable in the field. I liked to call him the Indiana Jones of field biology! In the evenings after supper with head torch and aspirator, it would be time to go on a Carabidae hunt.

He was also fearless, saving me from a pack of marauding peccaries in Ecuador, as well as rescuing me from certain death by bivouacking army ants in the dead of night as they surrounded our camp.

Terry was a modern-day Humboldt. It was his holistic approach to field biology with a deep knowledge not just of the insects of the rainforests of South and Central America, but also the flora and other fauna that enabled him to so clearly understand the relatedness of species, as well as the mechanisms of the forest that drives such incredible diversity. He was absolutely stoic in his defense of his estimate of 33 million species of insects – “an even higher number” he would say! And, having been in the field with him, with his meticulous observations of the microverse, his pioneering investigations into the forest canopy, well, I absolutely believed him. These were not assumptions from a data set or a modelling outcome. These were from direct in-field observations: a true naturalist.

My time with him was challenging, educational, inspiring, terrifying, motivating, demotivating, awkward, funny, curious, adventurous and time consuming. He was and is the reason I endeavor to be a good field biologist. His compassion, consideration and genuine every-day awe for the natural world is a method to live and work by. I will miss his shaggy dog tales, the most welcome cold beers and then later, martinis after emerging from a month in the field. I will miss his exacting method of teaching. For example, the second time I was in the field with him, with a few years in between, he grilled me on the species of trees we passed by along the path; which he had so carefully taught me on the previous trip. As a teacher and a mentor, he encouraged all his students to do better. He always displayed an exacting joy at imparting his hard-earned knowledge, knowing it was landing on fertile ground.

He talked often of his ‘academic father’ Dr George Ball, and in turn Terry became my academic father and George, my grandfather. He understood clearly the necessity for academic succession and the need for academic generosity in imparting knowledge to the next generation of coleopterists. This was not only the right thing to do, but also necessary for the continuance of the discipline of taxonomy to which he devoted his life. This generosity reached countless students over the years, particularly from Latin America. He was an able and creative academic supervisor and mentor; as well as quietly providing financial support to his students who could not otherwise afford to attend international entomological meetings. His legacy is in his teaching.

But, the trouble with Terry, was that he tricked us. We thought he would live forever. When on fieldwork in the primary jungles of South America whilst setting up collecting traps at 4 am, it was as if the rainforest were his orchestra and he the conductor. Canopy fogging is a race to finish before the dawn; it is wet and gloomy, muddy, and surprisingly cold. At 78, he was leading fieldwork expeditions as manfully as he was in the 1980s when he first began to develop these techniques of explorations into the jungle canopy. Not once could any of us younger than him complain. Because he was still getting the job done.

Although a great and long life, it was over too soon. There is still so much work to be done.

Whilst on fieldwork with Terry in his beloved Yasuní National Park of Ecuador, he would often ponder the wonder of nature. In his own words, ‘‘If you are studying biodiversity, how can you not study beetles?” And then more romantically, for he had a soft heart, “It’s just so obvious that there are as many critters in the rainforest as there are stars.”

As I embark on my carabidology path, I am lost without his guidance; and I miss him terribly. He is not gone though, he is still in my memory, ready with a black coffee, be it in the field or at his office at the Smithsonian, urging and inspiring me to dive head-first into a day of carabidology. RIP my dear Terry.

***Beulah Garner*** *is Senior Curator of Coleoptera at The Natural History Museum in London and was a friend and colleague of Terry Erwin.*

## Friend, colleague, and mentor

Like so many others, I owe the trajectory of my professional career to Terry Erwin. It was Terry who introduced me to carabid beetles as an accessible, diverse, and fascinating group of organisms. It was Terry who introduced me to George Eugene Ball, our shared mentor, professional role model, and friend. Terry gave me my first lessons in productive collecting and observational methods, which have produced abundant results for me over the years. He introduced me to the New World tropics and the amazing diversity he studied there and loved so dearly. He introduced me to bird-watching as a hobby to be enjoyed anywhere and anytime. But most importantly, he was my close friend and closest colleague for more than 50 years [ZooKeys 936: 149–152 (2020)]. I miss him intensely but also feel so grateful to have shared so much of my life with him.

***David H. Kavanaugh*** *is Curator Emeritus, Department of Entomology, California Academy of Sciences and was a long-time friend and colleague of Terry Erwin.*

**Figure:**
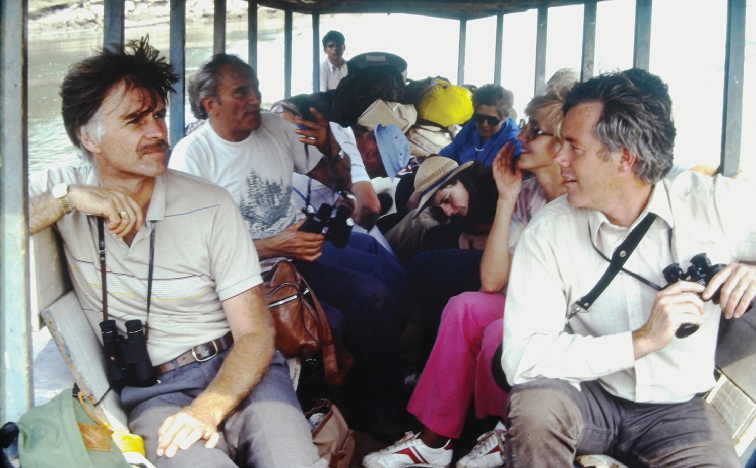
**Photograph caption.** Terry Erwin (L front) and Dave Kavanaugh (R front) en route to the Explorer’s Inn on Rio Tambopata, Madre de Dios, Peru. September 1984.

## Reflections on Terry Erwin, a caring mentor for the next generation of carabidologists

Terry was a wonderful and caring mentor who shaped the way that I view and think about science. He helped to introduce me to carabid beetle systematics, and to the world of entomological research. I started working with Terry during my second year of graduate school, after a year of collecting carabids in the cloud forests of K’osñipata Valley, Southeastern Peru. I had read many of his papers, and my Ph.D. advisor recommended that I take my collections to Terry at the Smithsonian National Museum of Natural History; of course, Terry was eager to help me learn about all things Carabidae. I was ready to learn, and a wonderful mentor-mentee relationship was born.

I remember my first trip to an Annual Meeting of the Entomological Society of America (ESA) with Terry, the 2008 meeting in Reno, Nevada. This was shortly after meeting Terry and was my first trip to a professional conference. Terry encouraged that I not only attend the ESA’s Annual Meeting in Reno, but he also helped plan a stop in San Francisco, California, to meet David Kavanaugh at the California Academy of Sciences. This is what Terry did for young carabidologists, help them network with the other giants of the field. After our stay in San Francisco, Terry and Grace had rented a car to make the journey to Reno; I can remember Terry reminiscing about growing up in Vallejo as we passed that exit and commenting on the incorrect Anglicized pronunciation of the Spanish word “Vallejo”. I can also remember driving over the Donner Pass in the Sierra Nevada Mountains and learning the story of the Donner Family from Terry. Looking back, it was so generous of him to work so closely with not just me, but many students. He always took the extra steps to teach the next generation of carabidologists.

Terry also served on my Ph.D. advisory committee at Wake Forest University. I always appreciated his attention to detail as an advisor. I remember well that when it came time to work on final edits of my dissertation, his meticulousness was even more helpful. I would make sure to get drafts of my dissertation to him by 3:00 pm, happy hour on ’Terry time‘. Terry lived in the D.C. suburbs and would start his workday at 6:00 am to avoid the traffic, and he would sit down with his evening martini by 3:00 pm. It always amazed me how well he could search my written works with a fine-toothed comb, paying special attention to typographical errors and consistency in formatting, not just the content. This attention to detail is so important, and to have someone like Terry be patient with my writing was so meaningful.

Terry’s mentorship has influenced my career as a teaching professor. Terry was an amazing carabid beetle taxonomist and systematist, but he was also an excellent mentor and teacher to the next generation. His lessons on carabid morphology and taxonomy extended beyond fieldwork and research and have shaped my own teaching philosophy and idea of what it is to be a good mentor.

***Sarah Maveety*** *is an Assistant Professor of Biology at Brevard College in Brevard, North Carolina. As a graduate student, she studied carabid beetle diversity in Peru; through this work, Terry served as an invaluable mentor and friend.*

**Figure:**
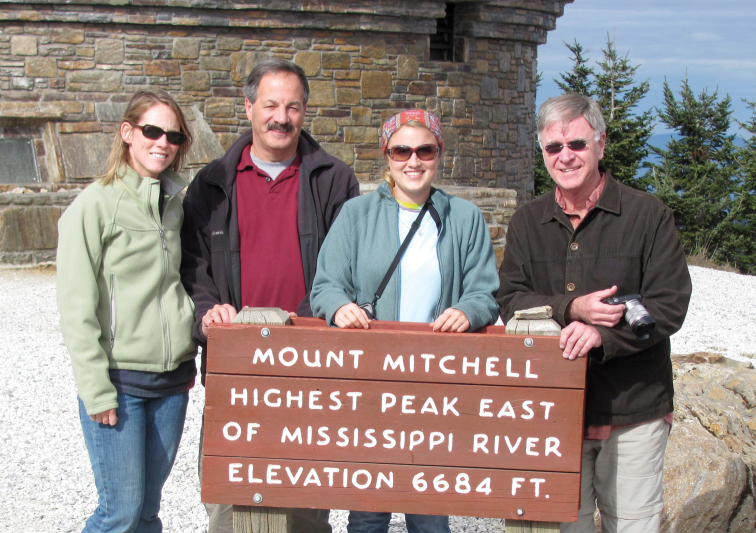
**Photograph caption.** Terry Erwin with his mentored students and Bob Browne (Professor of Biology and student advisor at Wake Forest University, Winston-Salem University, North Carolina) (L to R) K. Riley Peterson, B. Browne, S, Maveety, T. Erwin. Atop Mt. Mitchell, NC. November 2010.

## From the Smithsonian to the Amazon and Back Again

As a scientist, Terry was drawn to grand questions: How many species are there on the planet? What are the drivers of species diversification on a global scale? What is the turnover rate of tropical diversity? Is it possible to publish those descriptions quickly by establishing a journal that makes the publication process smoother and faster? Is it possible to describe every species of *Agra*!? Is it possible to write a series of books that describe every carabid beetle in the Western Hemisphere? Is it possible to shift the gender balance in systematics by promoting and empowering women in that field? Terry was a big picture guy, with splendid ideas and energy to push for positive change. Because of this, he is among the most famous entomologists of modern time.

As a human being, perhaps Terry’s most venerable attribute was to live completely true to himself, a self that was full of energy, joy, and kindness. He lived a life true to his inner spirit. Without a doubt Terry Erwin was one of the most honest people I have ever known. Terry listened to a drummer who beckoned him dance, and dance he did! His insatiable optimism, quest for adventure, and excitement about the future made him an absolute pleasure to be around. He honored his friends, and he found great happiness in the success of his friends and colleagues. He was genuinely kind to all and consistently faithful to his innermost nature. He brimmed over with a love of life, nature, the tropics, carabids, and fun times with friends and colleagues. It is a peculiar and perplexing feeling to lose a friend who seemed larger than life itself and I still cannot believe that he is gone. When I think back on all the times we shared, from being on a canopy fogging fieldtrip to Tiputini, to working on carabids during my annual visits to the Smithsonian when time stood still for weeks on end, to countless ESA and Carabidologists’ meetings where he seemed the happiest, to his many visits to Tucson (Arizona) – many stories come to mind.

Here is one from a visit to Tucson that he recounted many times. My husband and I were teaching a course on biodiversity for Columbia University at the Biosphere 2 Center, and we invited Terry to come out and talk to our students about his seminal multi-decadal research on insect biodiversity in the Ecuadorian and Peruvian Amazon. After delighting our students with stories of tropical diversity and fogging the rain forest canopy, his tone turned dark as he told stories of oil prospectors and hectares of palm plantations, ending his talk with a dire warning and plea for humankind to change their ways before we reach the point of no return. He dramatically exited stage right in silence, head bowed, with his final slide, a red Earth, burning its way into our collective memory. After his dazzling talk, the story Terry liked to remember began. We drove to the tiny old mining town of San Simon for a Mexican dinner, where he ordered a margarita that was served with great panache in a ’glass‘ the size of a fish bowl. Unfortunately, I don’t have an actual photo of him with that gigantic margarita, but when I think of him now I can see him there, his face blushed with a big smile animated by friendship, joy, and the ridiculous enormity of that margarita. We tried to recreate that scene many years later during one of my visits to Washington (see photograph). And while they were pretty good, those modest Washington margaritas were but a pale shadow to the aquarium-sized San Simon libation he kept alive in his memory, and nurtured as it grew in proportions over the years.

Terry was a positive force in my life and he left me with a deep gratitude for his friendship, a great respect for his brave and kind way of being, and a bugle call to action that still rings in my ears.

***Wendy Moore*** *is an Associate Professor and Curator at the Department of Entomology of the University of Arizona in Tucson. Wendy was a mentee, friend, and long-time colleague of Terry Erwin.*

**Figure:**
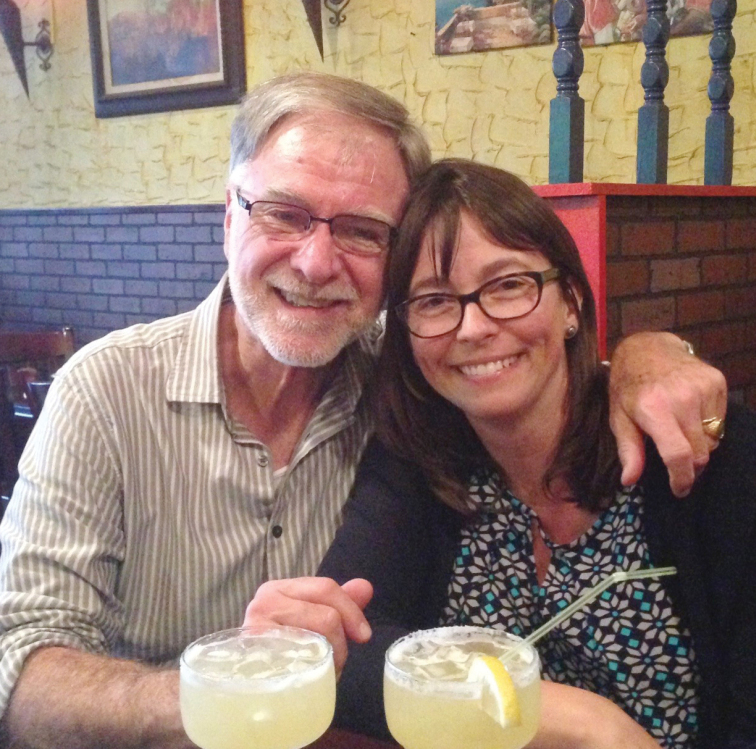
**Photograph caption.** Terry Erwin and Wendy Moore celebrating friendship in Washington, D.C. Photograph: B. Gravely, 2001.

## A kind person and most inspiring naturalist

When I had just graduated from college, I had the chance to meet Dr. Terry Erwin in a field biological station in the Ecuadorian Amazon. I remember being amazed by a talk he gave one night, explaining his revolutionary theory about how the actual number of species in the canopy of the rainforest could be way higher than we previously thought, and therefore, how that changed everything we knew about the number of species on the planet. A few years later, when I was exploring the canopy tied to a rope, I would always remember Terry and his explanations about life in the canopy. Thanks to him, now we know that a vast majority of the species in the tropical forest live there.

At that time, I was lucky enough to go with him to the field and assist him in his fogging sessions to collect the canopy fauna. I was new to the Amazon Rainforest at that time, and on the way to the fogging site, I remember him pointing out interesting species that caught his attention. “You hear that? That is a Screaming Piha, it is such an extraordinary bird, its call is very loud and can reach more than 100 decibels”. “Be careful, there is a spotted toad on the trail, don’t step on it”. In only a few hundred meters with him in the forest, I learned more about the jungle than in all the classes I had taken in college. His knowledge about insects and beetles was unique, but he knew pretty much about everything.

I saw him again a few years later, when I was managing the Station where I met him. He was always kind and happy to explain anything you would ask him. He always had a smile and a joke, and I always wanted to sit next to him at dinner, just so I could hear his stories, learn from him, and absorb some of his knowledge. We could have conversations about anything, from politics to pollination, and his expertise was always astounding. He was very pleasant and had more energy in the field than most people I know. We would even have conversations over email once his field season was over. Terry was always kind enough to ask about my life and ask if I needed anything. The times at the Station when Terry was there were precious. It was clear that he had an incredible passion about the rainforest and all the creatures that live on it. Many students visiting the station had heard of him and saw him as a sort of God of the insect world. But once they talked to him, they realized that despite his incredible reputation, he was an easygoing and kind person. It was always a pleasure to talk to him.

I was shocked when I heard the news about him passing away. Only a few weeks before we had had an email conversation, and he mentioned that he would come to the rainforest soon. I just could not believe he was gone. Nonetheless, the Terry that I knew was larger than life and there can be no doubt that his legacy will remain in all the people that met him and all the students he mentored. Even though my career has nothing to do with entomology and the fact that I had little interest on insects at the beginning, Terry fostered my interest in them, taught me to look at them from a different perspective and appreciate them beyond their looks … and to appreciate the variety of those looks as well. Now, when I see something, I inevitably ask myself, “I wonder what Terry would think”. I remember Terry not only as an amazing human being and good friend, but as a mentor and as an example to follow. He has and will be missed, but his true generous spirit will always remain with all who had the privilege to know him.

***Diego Mosquera*** *is a researcher in tropical ecology with Universidad San Francisco de Quito, Research Coordinator of the Tiputini Biodiversity Station in the western Amazon and Researcher at Instituto Biósfera. He and Terry Erwin were friends and colleagues for many years.*

**Figure:**
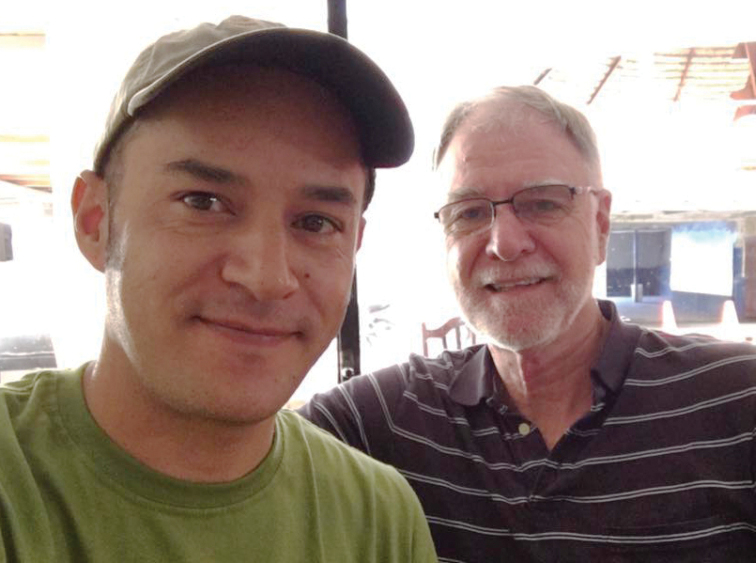
**Photograph caption.** Terry Erwin and Diego Mosquera having breakfast together just before the long journey to Tiputini Biodiversity Station. Coca, Ecuador, 20 June 2016.

## How *ZooKeys* was born

I first met Terry at the Third International Congress of Carabidology in Kauniainen, Finland in 1995. The Congress organizer, Jari Niemelä, who had spent a two-year postdoc in Edmonton, made significant efforts to diversify the profile of the attendants beyond the normal scope of the European carabidologists’ meeting (ECM), by inviting a number of prominent carabidologists from the USA and Canada, including George Ball, Terry Erwin, Dave Kavanaugh, John Spence and others. A fantastic outcome was that soon after the political changes in the Soviet Union and Eastern Europe, we were able to meet in person colleagues known previously through ‘postal friendships’ and, in some cases, long-lasting and productive collaborations.

This is how we started the wonderful tradition of North Americans attending the ECMs. A memorable meeting was held in Blagoevgrad in 2007, which was visited by a distinguished group of carabidologists from the USA and Canada, led by George Ball. Terry was also there with his wife Grace, while John Spence even brought four of his talented students in carabidology. The Blagoevgrad ECM launched a great tradition and an even larger North American contingent attended the meeting in the Dolomites, organized with much love and care by Roberto Pizzoloto! Although it was a wonderful meeting, we feel much regret and sorrow that it would turn out to be the last ECM for Terry. I have fine memories of meeting Terry, often in the company of Grace, during the past three entomological Congresses in Durban (2008), Daegu, South Korea (2012) and Orlando (2016) and at annual meetings of the Entomological Society of America, e.g. San Diego (2007) and Reno (2008). Along with improving the quality of the science, international meetings provide opportunities for development of mutual understanding and friendships born of common interest.

The meeting in Blagoevgrad was explicitly organized under the ’The East meets the West‘ slogan and, therefore, various parties, in addition to the sessions themselves were central to the experience. The post-meeting party that I hosted in the barbeque place at my house in Dragalevtsi near Sofia was a prime example. Among other things, I proudly offered my father’s home-made apricot rakia (an elixir best, yet not really accurately, translated as ’brandy‘ or ’schnapps‘) to my dear guests Terry and Grace Erwin, John Spence, Hans and Annelies Turin, Wifried and Doris Paarmann, Achille Casale, Dietrich Mossakowski, Thorsten Assmann, and others. It was a splendid evening and we concluded that we had just enjoyed one of the most successful carabidologists’ meetings ever. It seemed that it included the highest number of participants to have attended an ECM, with registrants numbering approximately 90. The resulting camaraderie and feelings of accomplishment undoubtedly led to the somewhat higher consumption of drinks. Or was the apricot rakia itself the inspiration? It certainly became a favorite of many.

At a quite late point in the extended evening, someone mentioned the name of an online and highly successful journal in taxonomy. At that point, Terry jumped in to say: “It’s definitely a nice and valuable journal, but we are somewhat fed up with it. It takes too long to publish there, the journal is swamped with manuscripts and the production quality could be better. It would be good to have a rival journal that focuses on online publishing, but performs better!” In a moment I responded to Terry: “Well, we have in fact been seriously thinking about launching such a journal and even have the name for it: “ZooKeys”! Would you like to become its founding Editor-in-Chief?” As one can imagine, the atmosphere at that point of the night encouraged quick and bold decisions. Terry answered: “But of course! Let’s do it!” In retrospect, as spirited this decision seemed to be, it turned out to be very sound!

On the next day, a small part of the ECM group left Sofia for the post-meeting tour. Our first stop was Bachevo, a village in the Razlog valley, situated between the Rila, Rhodopi and Pirin mountains, some 170 km south of Sofia. This was the place where we had previously celebrated the publication of our “European *Carabus* book” four years earlier. I remember showing Terry and Grace the beautiful landscapes of Perivol in the Rila Mountains, and relating a tale from the previous gathering in 2003. I recalled that George Ball had ’disappeared‘ in the bushes for several hours, determined to find the quite rare Balkan endemic genus *Xenion*. Funnily enough, it was just as Hans Turin, Achille Casale, Augusto Vignia Taglianti and Jose Serrano and myself started to worry about George and were discussing a ’rescue‘ operation for him that he emerged in full spirit out of the woods, carrying – believe you or not! – a *Xenion* in his hand! Curiosity and dedication to carabidology had deep roots in Terry’s academic family!

The evenings in Bachevo, especially in the company of my local friends are usually spent partying, and that one, in the presence of dear guests from abroad, was no exception. In the daytime we did not speak about “ZooKeys”, but it did not take us long to resume the conversation after the second round of rakia in the evening, spent at the nice terrace of the local taverna “Хитър Петър” (“The Smart Peter”). We discussed how to make “ZooKeys” innovative enough to change the landscape of taxonomic publication. Terry was extremely forward-thinking, and excited about the opportunities offered by the Internet to biodiversity scientists to efficiently publish revisions, checklists, and new species descriptions online that would be openly accessible to everyone, everywhere in the world. Many readers will remember his online catalogue of the North American ground beetles was one of the very first digital publications of this kind.

On the third evening, we gathered at the Orphaeus place near Devin in the Rodopi Mountains. During the day, while we were hiking around the spectacular landscape, Terry caught a *Bembidion* specimen by the river. When I asked him why he did not collect any more, he told me: “Actually, it is fine to keep just one as a precious memory from this fantastic place. I do not want to kill more beetles from here.”

Again, we did not even mention “ZooKeys” until the sun went down. I think that Terry and I had already begun to realize how much work we would face in launching and building this new journal from scratch. However, once more, the rakia did its job and we were undeterred. Later that night, as we sat at the terrace overlooking the river, we came up with concrete plans about who we should invite to sit on the editorial board, and where we could find the first manuscripts of stature sufficient to inspire interest in the journal and the key aspects of the journal’s philosophy, focus and scope that we wanted to emphasize in the opening editorial paper.

Although the above may lead some to conclude that your beloved journal, “ZooKeys”, was born in a frivolous, poorly considered, and rakia-infused discussion, it is my recollection of how it all started. A half year later, at a working breakfast together at the ESA meeting in San Diego (2007), Terry finally confirmed his agreement to become Editor-in-Chief of “ZooKeys”. And, just six months later, our opening editorial paper and the first “ZooKeys” articles had already appeared. Working with Terry was like that. The difficult became easy and significant accomplishments flowed while friendships were developed and the pleasures of apricots were enjoyed.

Today, “ZooKeys” is the world’s second largest journal in taxonomy and is considered as an advanced effort that actually changed the way of publishing in biodiversity science. Not so long ago, “ZooKeys” published its 1000^th^ issue (blog), after twelve fantastic years, more than 5,500 articles have been published and more than 12,000 species have been described as new to science! Also, I am really happy that “ZooKeys” published a Festschrift paper on the occasion of Terry’s 75^th^ birthday in 2015 (ZooKeys 541: 1–40. https://doi.org/10.3897/zookeys.541.7316), where in addition to biographic notes, we put together Terry’s complete bibliography and list of taxa named after him. In that year, “ZooKeys” also re-published Marlin Rice’s marvelous story about Terry’s career and leadership in entomology (ZooKeys 500: 9–24. https://doi.org/10.3897/zookeys.500.9772).

So, this is how it all began.

Thank you so much, our dear Editor-in-Chief!

***Lyubomir Penev****is the Managing Director and Founder of Pensoft Publishers and a Professor of Ecology at the Bulgarian Academy of Sciences, Sofia. He has worked actively with Terry Erwin to found and promote the journal* ZooKeys *and they developed a close friendship*.

**Figure:**
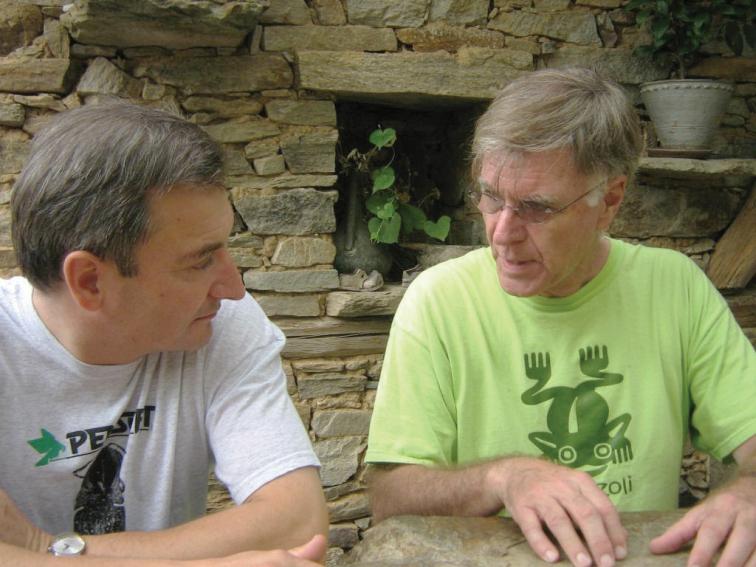
**Photograph caption.** “ZooKeys” will fly! Lyubomir Penev and Terry Erwin. Photograph: V. Peneva.

## My Neotropical companion: a friend and guide to the rainforest

Terry and I walked along a narrow trail in a floodplain forest of the Yasuní area in the Ecuadorian Amazon, and, as we walked, we scanned the ground and vegetation lining the path. We hoped to see a carabid beetle scurrying along the forest floor or the vegetation. The forest canopy, tree branches and lianas towered above, blocking the view of the night sky, our headlamps the only source of light in the dark, humid forest understory. Finally, seeing a beetle, I quickly grabbed it and looked at Terry for what to do next. Pointing to his arm, he said, “throw it in the beetle trap.” The ‘beetle trap’ he referred to was, in fact, the long hair that covered his forearm. I tossed the carabid on his arm where it struggled to navigate its way through the long hair. He responded jokingly, “the one pro of being as hairy as a bear.” We both leaned in to examine the tiny black beetle more closely. After only a few seconds, Terry spouted-off a genus name (one I had never heard of) and we popped it into a vial of 95% ethanol. That was it, my first neotropical carabid, but thankfully the first of many.

I recall the sublime feelings of excitement and joy from my first days in the Amazon. It had always been a dream of mine to spend time in the rainforest, so an opportunity to spend months in a megadiverse part of the Amazon was more than I could have asked for. It was 2011 and I was starting a project for my dissertation to study diversity and community structure of Carabidae at Tiputini Biodiversity Station (TBS), a remote field station immediately adjacent to Yasuní National Park. Who helped me plan and organize this project? Only the most qualified neotropical carabidologist in the world, Terry Erwin. The ‘Beetle Man’, who had at least 30 years of neotropical Carabidae experience, helped me get to know the forest, its fauna, and of course its carabids. He ensured all the sampling materials I needed made it to the field station, walked through the forest with me to select my sampling sites, assisted with the assembly of my first flight intercept traps and he even helped me set up pitfall traps, after warning me several times that they were not an efficient sampling technique in neotropical forests. Not surprisingly, he was correct.

During another night of carabid collecting at TBS, we were wading through a small body of water in the terra firme forest when we noticed a set of yellow eyes, watching us from a distance. Whatever it was, it was far enough away not to be of immediate concern and Terry reassured me it was nothing to worry about, so we continued collecting. As time passed, we noticed the eyes were changing locations, seemingly getting closer each time. Terry and I reconsidered our level of concern, but decided it was most likely a mammal of some sort and still *probably* nothing to worry about. We continued collecting. Then, the eyes appeared stone’s throw away from us and we watched as they silently slid into the water, the same water we were standing in. As it did this, we could just barely make out the faint outline of a very large caiman, whose glowing eyes remained fixed on us. With that, no additional discussion was needed. Terry and I swiftly scrambled to the opposite shoreline. Once we were a safe distance away, we laughed, checked our pockets to make sure we still had our collecting vials and headed back to the cabana.

These are just two memories with Terry while in the Amazon. During the several weeks we were both at TBS, we walked the trails and sorted specimens under the microscope for hours, meanwhile chatting about beetles, biodiversity, fractal universes and life in general. He was a great story-teller, whose stories would captivate any audience and have everyone eagerly waiting in anticipation for what was coming next. His energy was infectious and encouraging; he was an inspiration to many. Field days with Terry will forever remain as some of my fondest memories. He was so ’at home‘ in the rainforest and never hesitated to share his passion and knowledge of it with anyone he met. He introduced me to many of the Amazon’s plants, insects and other arthropods, birds, and mammals. The main results from my project are published in this memorial volume. It is the most important work that I have ever been a part of and it is an honor to have coauthored this paper with Terry.

Terry was a member of my PhD committee, a mentor and most importantly a friend. He helped to guide me through my graduate school years and was a constant presence for support, both professionally and personally. I am grateful for the time spent with him and the opportunities he provided. He did everything he could to bring us all into ‘the fold’ and introduce us to as many opportunities as possible. There is no question his guidance and friendship shaped how I see the world and helped me grow to be the person I am today. We shared many laughs (and drinks) during the 12 years we knew each other. I am incredibly fortunate to have had the chance to learn from and work with Terry Erwin. He is missed; but his legacy lives on. Until we meet again, abrazos, my friend.

***Katie Riley Peterson*** *is an Assistant Professor of Biology at Pfeiffer University who studied tropical ecology with Terry Erwin in Ecuador. Terry was a mentor and good friend for more than a decade.*

**Figure:**
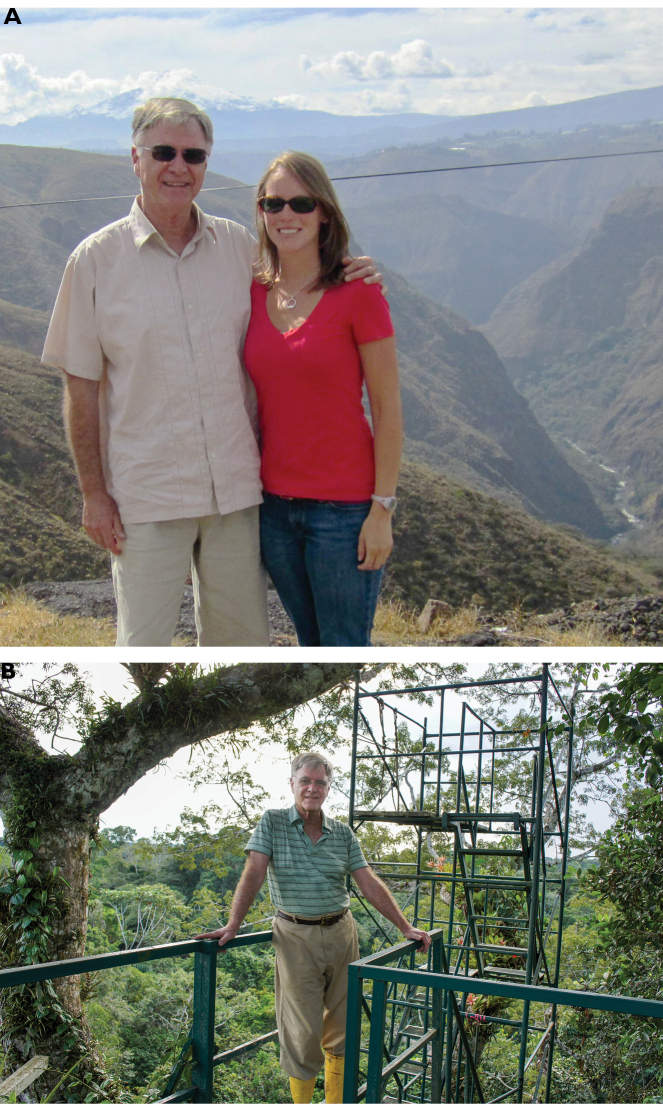
**Photograph caption.** Memories of work in Ecuador **A** Terry Erwin and Katie Riley Peterson enjoying a scenic stop en route to the Mercado Artesanal at the Plaza de Ponchos in the town of Otavalo in the Andean Highlands. 17 June 2012 **B** Terry on the platform of a forest canopy observation tower near Tiputini Biodiversity Station. April 2011. Photograph: K. Riley Peterson.

## A passion for high quality presentations, and understanding biotic diversity in the natural world

I met Terry Erwin in 1966 when he was a PhD candidate at the University of Alberta and I was but an undergraduate. Most students in the Department of Entomology at Alberta at the time were MSc or PhD students; however, we few undergraduates interested in entomology were treated like graduate students. Terry took me under his wing and provided advice on public speaking, explained the joy of unravelling complexities in the natural world and showed me the importance of understanding the distribution and inter-relationships of flora and fauna. Terry’s mentorship profoundly impacted my career. Today, 55 years later, I still think back fondly to his teaching and credit him with key aspects of my own approach to entomology.

Terry was part of an enthusiastic bunch of graduate students and I often ate lunch with three of them: Terry, David Larson (of carabid and dytiscid fame) and Robin Leech (of spider fame), who shared a small room in the basement of the Agriculture Building. I was welcomed to participate in their discussions on all topics – generally entomological or natural history more broadly. Their stories of field work and collecting expeditions captured my young imagination. Terry in particular talked about taking field trips to exotic places because, as Charles Darwin and Alexander von Humboldt understood, work in far-flung locales attracts attention from other scientists and the public.

Thus, when an opening came up for an assistant on a 1967 expedition to Lake Hazen in northern Ellesmere Island, about 800 km from the north pole, Terry encouraged me to apply for the post. I was chosen and to this day I relish the expedition that led to speaking engagements, and career-long interest in understanding the complex life histories and strategies of northern flora and fauna.

Upon my return, I accompanied Terry on a field trip to the Department’s George Lake Field Station, ca. 80 km northwest of Edmonton. We found cynipid galls induced by *Diplolepispolita* on wild roses and Terry quipped that here was an ideal MSc project. He suggested that if I could figure out how cynipid wasps made galls, I could help decipher one of the most complex insect-plant relationships in the natural world. This became my MSc, but little did I realize, I would be following Terry’s suggestion for the rest of my career.

Terry encouraged me to include the botanical side of gall studies in my research on cynipids so I went to the University of Saskatchewan to study with both an entomologist in community ecology and a botanist in plant development. The galls of *D.polita* from George Lake were among those induced by six species of *Diplolepis* found in Alberta and Saskatchewan that I studied to earn my PhD. It would remain a favorite research subject of mine (e.g., Memoirs of the Entomological Society of Canada 165, 139–163 (1993)).

As it turned out, the gall of *D.polita* exhibited both the complexity and an intriguing distribution, as Terry predicted back in 1967. He was right to suggest galls both as excellent subjects for studying complexity in the natural world and the value in using both entomological and botanical approaches in exploring how cynipids gain control of plant growth (e.g., Canadian Journal of Botany 84: 1052–1074 (2006)). As a tribute to Terry, I illustrate here the George Lake gall (Photograph a, b), that he introduced me to in 1967.

Working with students in my laboratory, I went onto show that inquiline cynipids of the genus *Periclistus* attack and remodify galls of *D.polita* (Photograph c) and that parasitoids of the genus *Eurytoma* (Photograph d, e) attack both galls with only *D.polita* and those modified by *Periclistus*, consuming the larvae of both species before finishing their development by consuming gall tissues.

I also remember Terry explaining that one of the greatest rewards in science is to stand in front of your peers and explain your discoveries. Terry had taken a course in California on public speaking and offered to teach me the basics. For example, he listened and commented upon several versions of my presentation describing life history strategies of George Lake gall wasps before I presented at the 1968 annual conference of the Entomological Society of Alberta.

Terry held that a presenter of public and scientific lectures was akin to an entertainer because both strive to keep an audience’s attention for a set period of time. He stressed the importance of deportment, being smartly dressed, using an appropriate number of high-quality slides, and practicing to stay within the allotted time. “Command your space”, he told me, “be aware of your body language and make it look like you are enjoying yourself. Make eye contact with as many people as you can because this demands audience attention and keeps the focus on yourself and your message. Speak in simple sentences and don’t show off your expertise by using specialized vocabulary. Let your personality shine through; your audience will trust what you have to say if they can see you as a real person”. Readers of these words who have heard Terry speak will know he was among the best at commanding the attention of his audience. His words of wisdom remain applicable today with PowerPoints and virtual presentations and they provided the core of advice that I passed onto my own students.

Terry stressed the importance of depositing voucher specimens at public institutions and how this effort would inevitably lead to further scientific investigation. Deposition of vouchers became my habit, something encouraged in my own students and it has been rewarding to see this material used by others to solve interesting problems.

Even back in 1967 when he himself was a graduate student, Terry understood the importance of stimulating young minds. As reported in one of the tributes to his legacy (https://ibol.org/barcodebulletin/illuminations/30-million-reasons-you-will-be-missed/) “Terry understood the importance of nurturing the next generation of talent, and especially the importance of diversifying the [scientific] pipeline. Terry liked to say that he plants seeds – ideas in students – and watches them grow. He planted countless seeds that grew strong and bright.” Incredibly, in the 1960’s, he was already expounding on the importance of biologists studying factors impacting biodiversity, even among gall communities, well before he became world famous by using his knowledge of carabid beetles to mold our thinking about protecting biodiversity. I am forever grateful for being one of the first of Terry’s ‘seeds’, and to have benefited from his encouragement, guidance and introduction to a life and career devoted to studying the natural world.

***Joe Shorthouse*** *is a Professor Emeritus at Laurentian University and a long-time friend of Terry Erwin.*

**Figure:**
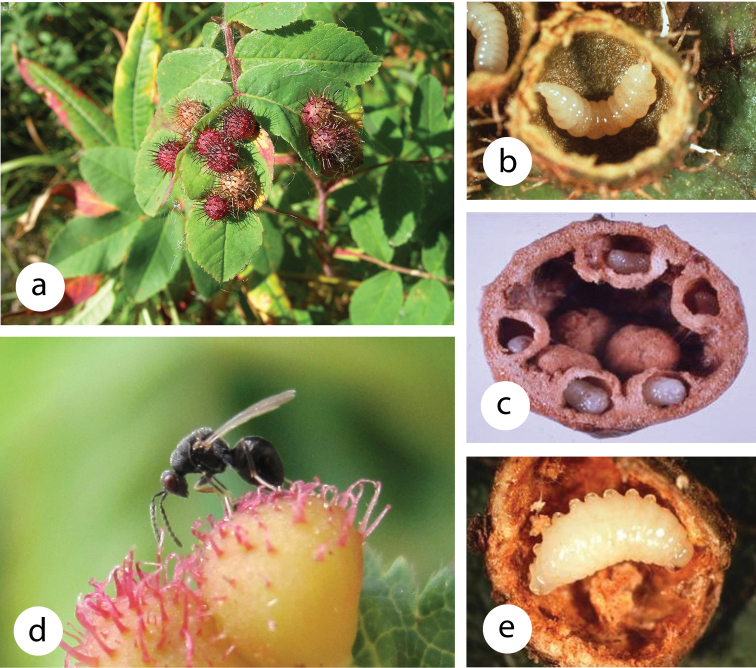
**Photograph caption.** Galls induced by the cynipid *Diplolepispolita* found at the George Lake Field Station and associated species **a** Maturing galls on the leaves of *Rosa acicularis***b** Larva of *D.polita* in dissected gall **c** Larvae of the inquiline *Periclistuspirata* that had structurally modified a gall after killing the inducer **d***Eurytomalongavena*, one of five species of parasitoids that attack gall inhabitants, ovipositing in a gall of *D.polita*. **e**) Larva of *E.longavena* after it had consumed larvae of gall inhabitants and gall tissues.

## Terry Lee Erwin, where do I begin?…

Boss, mentor, and more importantly, friend for over 40 years. We spent many mornings over coffee, trouble-shooting the ’crisis du jour.’ Frequently, our conversations had me exclaiming, “You want to do what?”! He had the ’big picture.’ My focus was details and logistics. We would problem solve together, often thinking outside the box. We usually came up with a ’Plan A‘ and alternatives. We made a good team.

Terry always allowed me to make mistakes, then turned them into teachable moments. One of my first trips to the jungle was at the height of the rainy season. A meter of rain in 30 days left us hip deep in mud and water. In a ’Jane of the Jungle‘ moment, I grabbed a vine and tried to swing across a small creek. After I fell unceremoniously in the water and after the entire crew’s laughter subsided, Terry talked about the importance of lianas and their place in the ecology of lowland forests. A memory to this day.

Another memory. One Monday morning, I stomped into his office, telling him I had quit grad school. He let me rant for a while as I pulled out my first draft and what my committee had done to it. Once I had taken a few breaths, he shared some personal experiences he had in Edmonton and his shared frustrations. He calmly went through the paper, offered suggestions, and that night, I was working on draft #2. Again, a teachable moment.

I saw Terry’s professional strength and perspective one memorable morning. Just back from Peru, Terry calmly called me into his office, had me put down my coffee cup, and quietly informed me the Tambopata lab had burned to the ground. Virtually all equipment – gone. Many personal effects – gone. Years of data notebooks –gone (copies existed in my office). Gratefully, there were no injuries. After a period of shock, we went into overdrive to raise funds to replace the equipment and we did.

In the big picture, Terry taught me many things, but most of all, I learned about myself. Everything I became at the Smithsonian happened because he took a chance on a timid student who lacked expertise in entomology but who was pretty good with computers.

Terry, it has been a year since I talked with you, and I am still awaiting an email about the *Agra* project. It needs to be finished.

You may be gone but you are not forgotten. I miss you my friend,

Lindy

***Linda Sims*** *worked with Terry Erwin at the Smithsonian Institution where Terry was her boss, mentor, and friend for 40+ years*

## A mind for beetles and a heart for beetle people

Terry Ewin’s conceptual and practical accomplishments in both tropical biology and carabidology have been legion and influential. He dramatically increased understanding of the Carabidae and the availability of information about the taxon for enthusiasts. He was cast in a mold similar to that of his PhD mentor, George Ball. Their strongest influences were decidedly interpersonal and educational. Even though their respective academic work was prodigious its own right, their influences were multiplied by friends, students, and associates.

After finishing my B.A. in Biology, I went to the University of Vermont to study ecology. As time passed, my efforts at ‘save the world’ ecology were not going all that well, but I was enjoying weekend insect collecting trips with Ross and Joyce Bell. I first heard about Terry from Ross, who had been the external examiner for Terry’s doctoral dissertation at the University of Alberta. Ross recounted Terry’s interesting PhD work with *Brachinus* as we encountered *Brachinusfumans* in the field. That sort of work immediately struck me as an example of something that I would find more interesting than investigating water quality in Lake Champlain. The next week, I switched my program of study to entomology under Ross’ supervision. I had been caught, tempted in by Terry’s fascinating work!

My earliest personal contact with George Ball and his students came in 1972 at an entomological meeting in Montreal. I believe that Terry was away in Sweden on a post-doc with Carl Lindroth at the time; however, his visit with the Bells upon his return reinforced the view that the Alberta carabidologists were an interesting and enthusiastic bunch. I liked the thought of casting my academic lot among them. Soon both Terry and Dave Kavanaugh had become friends and inspirations, primarily through correspondence. This was encouraging for a youngster trying to find his way down the winding road of natural history into the domain of science. Such wanderings are best done in the company of an academic family. By the by, Terry and I became academic brothers and I often looked to him for guidance and advice in my work with carabids. By then, you might say that I had been self-identified and diagnosed! Among many good memories of Terry, I am perhaps most fond of two, one of formative events early in my career, and another that is relatively recent.

In 1975, after beginning study toward a PhD at the University of British Colombia, I received a message from Terry. It was as an invitation to speak about my MSc work at the First International Symposium on Carabidology to be held as a satellite meeting of the International Congress of Entomology in Washington, D.C. during the following summer. I was amazed and flattered to have received such attention, but doubted that, as a poor student, I could afford to attend or offer much at the meeting. Terry urged me to come and immediately offered to arrange funding through the Smithsonian. My plans were laid, and what a meeting it was! Terry and Linda Sims had arranged a spectacular session that included some of the world’s leading carabidologists. It was grand to present and discuss my own work at the meeting; however, the opportunity to interact with carabidological giants like Carl Lindroth, Phil Darlington, Russel Coope, George Ball, Ross Bell, and a host of very capable and enthusiastic young carabidologists was right over the top. I began to understand that human interactions can be hugely important in development of science, and that the zones of friendship and fulfilling science can overlap in a wonderful way. As a result, I am afraid, I was pinned and self-labelled! Terry’s enthusiasm for carabids and his interest in bringing young people of common interest into ‘the business’ was remarkable.

Work with carabid ecology and diversity became a main pillar of my own subsequent research. Four decades later in 2017, I was in Bogota, helping an entomological friend and a couple of her Colombian graduate students with biodiversity projects in the Oronoquia region of NE Colombia. By this time, incidentally, I had come full-circle back to ecology aimed at saving the world by following Terry’s lead with respect to the biodiversity crisis. The identification of our Colombian beetles using Hans Reichardt’s key to neotropical genera sailed along until eventually I was stumped by specimens representing six different morphospecies. So, I sent Terry an email note describing the beetles and asking for his help. Within moments he had come back. Unfortunately, band width was too slim to make video chat work, so we resorted to text exchange and photo attachments. A 3-hr back and forth was on! Terry was at home in Washington, but right out of his ’mental taxonomic key’ he led us to solid generic names for five of our problem taxa. As far as we knew, there were no published keys yet available for the carabid fauna of Oronoqia that would be better than Reichardt’s. However, when I described the sixth group of specimens and sent a general habitus picture, Terry replied that I would not find anything like that in key … because he was just then describing the genus as new! He was pleased to have the first record from Colombia. We exchanged some email smiles and ‘Ha-Has’. As I had long understood, Terry was a master of his craft and most generous about sharing the knowledge that he was creating. This attitude of his has been foundational to progress in understanding the carabids of neotropical areas.

Terry will be sorely missed but we who knew him will remain grateful for his influence on our lives and our field of study. As discussed with several mutual friends on the sad day after his passing, we can now happily imagine Terry together with Carl Lindroth, Phil Darlington, George Ball, Ross Bell, Hans Reichardt, Piet Den Boer, Augusto Vigna Taglianti, Martin Baehr, Konjev Desender, Wilfried Paarmann, Jan Szysko, Shun-Ichi Uéno and others at a ‘big family’ party in some other dimension. The bar tender will be busy and there will be animated discussion about where they will go to investigate carabids when the bottles have been emptied. Friendship borne of common interest will be palpable and when they get to the site chosen, no stone will be left unturned … and now that Terry is there, we can be sure that ground beetles will fall from the tree tops as well! In the meantime, there remains work for the rest of us to do, inspired to carry on by the efforts of exemplary carabidologists like Terry Erwin.

***John Spence*** *is an ecologist, carabidologist and Emeritus Professor at the University of Alberta in Edmonton, Canada. He was a friend, colleague and member of Terry Erwin’s widespread academic family for nearly five decades.*

**Figure:**
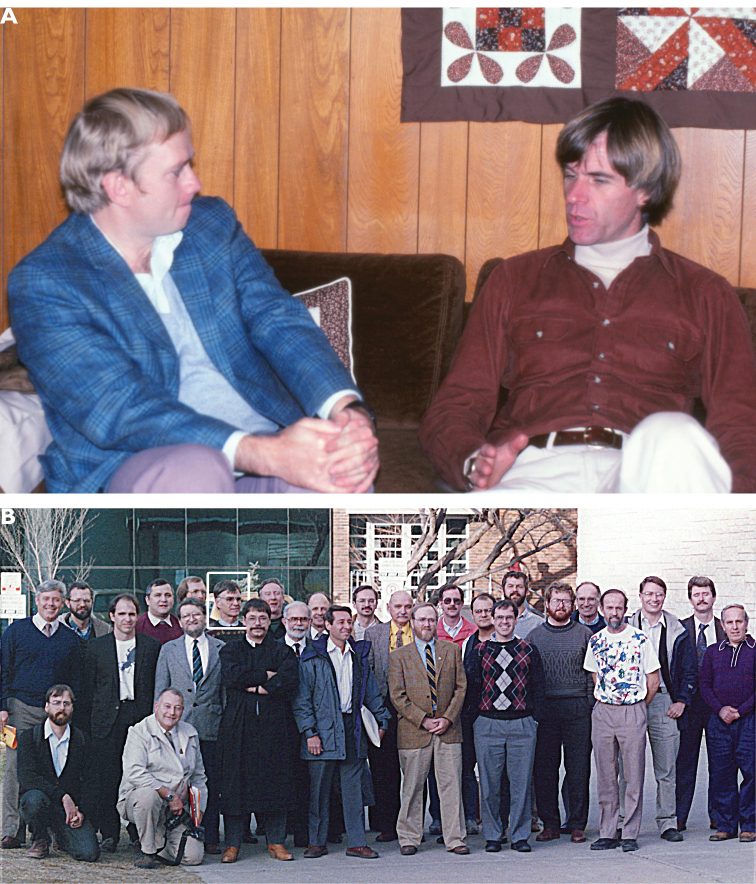
**Photograph caption.** Memories of Terry Erwin and his academic family **A** Earnest conversation between John Spence and Terry at the home of David and Bev Kavanaugh. December 1981. Photograph: D. Maddison **B** Terry (indicated in the white rectangle in left-center of the photograph) among the students of G. E. Ball on the occasion of Prof. Ball’s retirement symposium in Edmonton. Spring 1992. Photograph: courtesy of D. Kavanaugh.

## Being Terry’s Tech

He liked to quote William Beebe a lot, especially, “Yet another continent of life remains to be discovered, not upon earth, but one to two hundred feet above it.” It was probably the early 1970s when I first heard Terry Lee Erwin introduce a seminar with those words and continue to rave about the diversity of species in tropical forests. I was a student of entomology at the University of Maryland and starting work for a M.S., with a focus on beetles but with interest in learning to recognize and identify most all insects to family level. Because I liked the challenge of sorting mixed collections and delivering each lot to specialists for more study, hearing of Terry’s rainforest canopy samples planted a big seed. Given a few early contracts with the Smithsonian Institution and later being hired as a full-time technician, I found myself often immersed in Terry’s canopy catches, as well as similar jobs for other curators. And a number of travel opportunities came along ... little did I expect to make two visits to Kartabo Point, Guyana, the former site of William Beebe’s field station, collecting many things, including carabids for Terry! My best memories of Terry fit under the following four subheadings.

*Contagious Carabidosis.* Terry introduced me to many well-known and accomplished entomologists, naturalists, and colleagues, and I got to work with them in lab and field. Still humbled to have met his mentor George Ball, I remember well our expedition to southern Venezuela, where I got to fly by helicopter with George to the top of Neblina Tepui, to collect beetles. And at the basecamp, Terry gave a demonstration of the fogging technique, wearing that red bandana of his, as insects fell like rain. I did not take part in any of Terry’s major fogging trips, but at the museum, minded his catches and put in many hours sorting those rich lots, preparing and shipping specimens. Learning to sort carabid genera in the collection and in the field with Terry and George was an unexpected benefit that inspired me to publish a few new distribution records for both temperate and tropical species. Collecting ground beetles wherever I traveled soon became a focal point for me. Terry supported my work in many New World countries plus Malaysia, Madagascar, and in the Maryland back yard.

*In the Soup.* Many technicians, students and volunteers over the years found weeks of occupation in the sorting of Terry’s ’bug soup’, rich mixtures of many taxa usually preserved in alcohol, taken as ’fallout’ from pyrethrin foggings of forest canopy at dawn. We found many samples to mostly consist of ants, thousands in a single 125 ml bottle, usually with one dominant species indicating that a large colony had been fogged. This made sorting out specimens of other orders more tedious, first picking larger insects off the top, then the ants, and to the bottom ’residue’ where the smallest and often best species were. These included incredible myrmecophilous beetles unknown to specialists. These and other canopy finds, never collected before and likely never to be found again, continue to inspire systematists and generate descriptions of new genera. The ’arboreal ground beetles‘, especially the genus *Agra* Fabricius, got first attention.

Terry encouraged my long interest in the ’other ground beetles‘, Tenebrionidae, and so these were sorted out and many were dry-mounted for study. Diversity of darklings in the canopy is equally amazing to that of carabids. For example, we looked at members of the speciose genus *Strongylium* Kirby from a series of foggings in Ecuador; about 12 species are known north of Mexico, but from a single hectare of Ecuadorian forest, we found more than 85 morphospecies. Biodiversity at its finest!

Working for Terry was not always easy. *Agravation* Erwin, 1983 describes some of it. But we did find pleasure in coming up with names for our new beetles. The times of tending to non-entomological tasks such as ordering supplies, and training interns who had never handled insect specimens before, was frustrating, in particular when Terry was on extended fieldwork. But documenting beetle diversity was a perpetuating force.

*Latinos and Lebiines.* Terry was excellent in involving and training the resident talent of the countries that hosted his work. Museum and university coleopterists and beginning students alike were supported through his projects, mainly in the pursuit of those arboreal ground beetles. Many helped in the field with setting up the fogging sheets, collection of samples, and other services. Some were funded to travel to Washington and work in the USNM collection. Many became co-authors on papers with Terry, and some had species named after them. Terry liked the collaboration and camaraderie, and being in the spotlight with photographers and journalists that helped spread his biodiversity message. Bumper stickers that read “Save tropical forests...30 million insects can’t be wrong” were displayed for many years on road trips.

*Washington Biologists Field Club.* As fellow members of the WBFC we shared in the spring and fall social feasts on Plummer’s Island, on the Maryland side of the Potomac River, where Terry had earlier surveyed carabids for a much-cited paper in 1981. The club funded this study and continues to support research of students and members. The WBFC website (https://wbfc.science/single/?smid=437) has Terry’s autobiography, updated after his passing. Terry always invited guests to the events, and some got nominated for membership. Twice a year, on the ’work day‘ one week before the traditional ’shad bake‘ or ’oyster roast‘, his pattern was to hike to the island early (he was a morning person) carrying his broom from home to sweep the floor of the historic 1901 cabin. Early club coleopterists, e.g., H. S. Barber, E. A. Schwarz, and Henry Ulke walked that space, and sat by the fireplace, pinning beetles. Who will come to sweep those old planks now?

Motivation for biological conservation, both local and worldwide, owes much to Terry’s advertisement, and the excitement that he showed in any new discovery. Our note in 2007 on the first Maryland occurrence of *Phloeoxenasignata* (Dejean), a mostly tropical lebiine apparently moving north, provides one example. A few years later, he rushed into my office to show me a pinning tray with a few examples of this species captured recently in a Malaise trap on Plummer’s Island! It was a new record for this most researched island, not listed in his 1981 study. The age of discovery continues, and will go forth, including Terry’s many mentored followers and the great legacy of specimens left behind for future generations of systematists, conservationists, and those of us who just like looking at beetles.

***Warren E. Steiner, Jr.*** *is Research Collaborator at the Department of Entomology, Smithsonian Institution. He served as Museum Technician/Specialist for three decades (retiring in 2010), after many assignments under Terry Erwin, assisting in specimen collection and other museum research duties.*

## Terry comes to Yasuní

Literally in the middle of nowhere, Ecuador, I met Terry Erwin in an oil operation. It was January of 1994 and we had both been contracted by the Ecuambiente Consulting Group to evaluate the environmental impacts of a never-heard-of oil consortium, Maxus, in the concession area known as Block 16. But this was not just any oil field. This was Yasuní, the region expected to be the most species-diverse place on the planet because of its location in the western Amazon Basin near the equator and the Andean foothills. Due to the great likelihood of extreme species diversity, Terry saw collecting there not only as a golden opportunity, but very much a happy and fortuitous duty. Thankfully, most of the reserve was still completely intact, primarily because ’modern‘ humans had been excluded by ’the fiercest people in all of Amazonia‘, the Waorani, who had called the Yasuní home for millennia. The stakes were higher than high, for this unique indigenous culture and for nature. Justifiably, the eyes of the world were upon us, not only scrutinizing the oil company, but also the scientists who were there to ’oversee‘ the whole thing. As a newly minted PhD, I recognize that my inclusion as part of the environmental assessment team was quite serendipitous, mostly because I happened to be one of a miniscule pool of Amazonian fish specialists available in-country. In contrast, Dr. Erwin was there as a world-renowned researcher and conservationist who could lend real credibility to the science that was to document circumstances *in situ*, without being distracted by any controversy in the press. Neither of us would have chosen to be associated with the extraction of crude oil from a highly sensitive part of the Amazon, but like the other collaborating scientists, we saw this as an irresistible chance to spend some time in the legendary and difficult-to-access Yasuní Biosphere Reserve, and potentially, to strengthen arguments for conserving this pinnacle of nature with an overwhelming abundance of data.

Terry arrived at Ecuambiente’s field station, Onkone Gare, with a cohort of young, aspiring Ecuadorian biology students who were instantly converted into a well-oiled entomology machine fueled by pure curiosity. The ’Carmitas‘, as they came to be called, were out in the forest every morning long before dawn getting the day’s canopy fogging done and then, upon return, spent hours mesmerized by the riches of their collections. Certainly, Terry’s team, and everyone else in camp, was profoundly inspired by his unending drive for discovery, as well as being continuously animated by his wry sense of humor. One can only wonder how many Latin American entomologists over the last several decades might trace the beginning of their careers back to an interaction with Terry.

During this same phase of history, my home institution in Ecuador, the Universidad San Francisco de Quito (USFQ), was talking with esteemed Boston University bat biologist, Tom Kunz, about possible collaborations for research and study-abroad initiatives. Soon, I would be choosing a plot of land to establish the Tiputini Biodiversity Station (TBS) some 20 kilometers downstream from Block 16, in an even more pristine area, without road access. Because Terry had some long-standing queries about β-diversity and species turnover, it was not long before he started coming to TBS regularly to carry out treetop fumigations comparable to those previously made along the Via Maxus. What a privilege to have him at TBS so many times over the last couple of decades as he continued describing new species while generously sharing both time and knowledge, interacting with students, making research presentations on site and championing our cause back in the U.S. and beyond.

Whenever the calendar did not permit my being in the field with Terry, we would always get together for dinner in Quito to mull over science and life. Whether the venue was a nice restaurant or the Swing-Sempértegui home, Terry’s favorite drink, a dry martini, was always on the menu. And of course, like anyone who knows what he likes, he had a personal recipe perfected through repeated experimentation over years of excruciating sacrifice. Terry’s version of this classic cocktail starts with a splash of dry vermouth swirled around the glass and tossed out, leaving only a hint of perfumed influence on the subsequent pour of near-freezing Chopin vodka. A lemon twist is rubbed on the rim of the glass to finish preparation. No olives in sight.

The Erwin canopy collection methodology was also polished over many years of trial and error. A key part of the process is getting fumigations done while the atmosphere is cool and still, which is painfully early in the tropics. The fogger, a portable gas-powered thermo-nebulizer, is a beast of a machine reminiscent of some of the larger ’firearms‘ that show up in ’Men in Black‘ movies. Rather than having to transport this cumbersome piece of equipment each time, Terry would always leave it at our station between expeditions. On one occasion in the late 1990s, one of his Ecuadorian collaborators needed the fogger for a study in another part of Ecuador, meaning that it had to make its way back to Quito. The question was how. This instrument was considered somewhat vulnerable and impossible to replace in-country. The answer lay with one of my Boston University alumni who happened to be at TBS with a student group at that moment. Due to the size and weight of the apparatus, I rather sheepishly asked Dan Janes if he would be willing to lug the bulky device back to the capital city on his return trip. He immediately agreed to help and pointed out that my profuse gratitude was entirely unnecessary. He explained that for a biologist, conveying Terry Erwin’s fogger is on par with a chance to carry Elvis’ guitar! Indeed, the number of entomologists in all of history who have reached ’rock-star status‘ must truly be quite limited.

***Kelly Swing*** *is a professor of tropical ecology at the Universidad San Francisco de Quito, the founding Director of the Tiputini Biodiversity Station and a longtime friend and colleague of Terry Erwin.*

**Figure:**
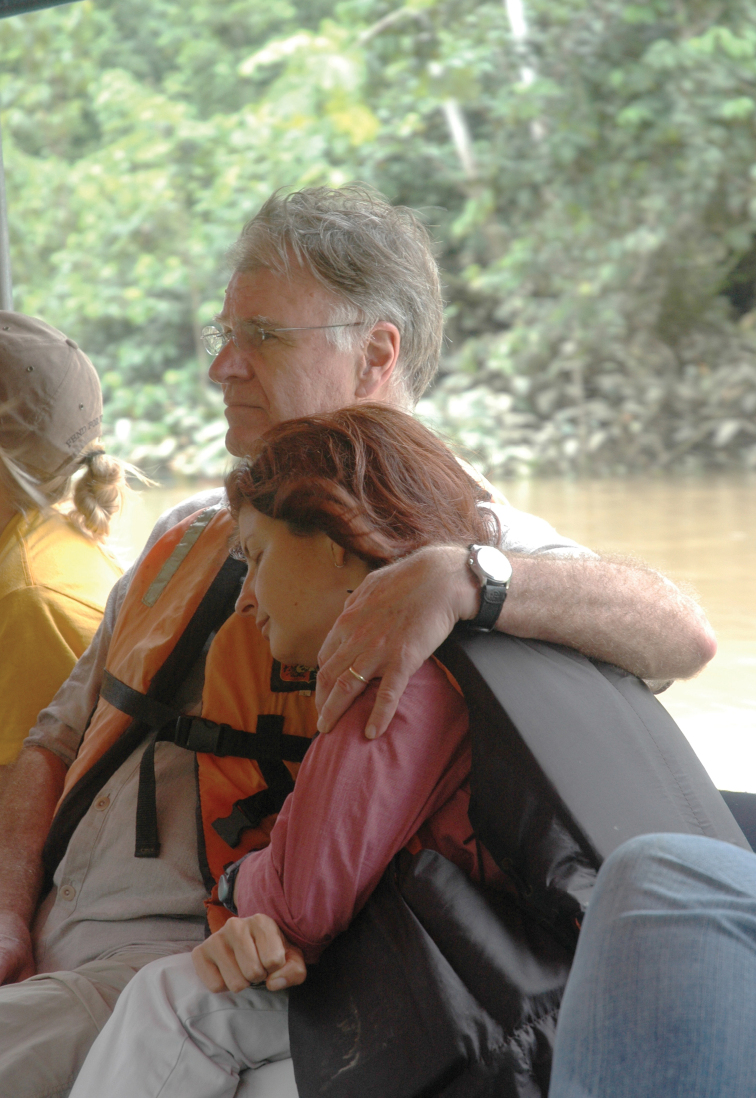
**Photograph caption.** Terry with his wife and Peruvian ecologist, Grace Servat, en route to the Tiputini Biodiversity Station along the Tiputini River, an important south bank tributary of the Napo in eastern Ecuador, 27 March 2008. Photograph: K. Swing.

## A Trans-Atlantic friend and carabidological bridge-builder

My first contact with Terry must have been more than 40 years ago when I was working on a compilation of all the literature that could help me create solid European distribution maps. When this project was about to be derailed by a then new checklist of European ground beetles, as a newcomer to carabidology, I turned for help to some great names in carabid taxonomy, like Carl Lindroth, George Ball, and Terry Erwin. I knew that the latter two, together with Donald Whitehead, had just published a new checklist in the North American Beetle Fauna Project (Erwin et al. 1977). I can’t remember the exact subject of my inquires and the snail mail letters have been lost over the years, but I do recall that as a newcomer to carabidology, I was amazed at the enthusiastic and friendly nature of the advice from Terry and others, that I received by return mail.

This of course had a very stimulating effect on my progress with the project. This enthusiastic support was most welcome, as the work had to be completed in my own time due to a reorganization at my institute, where the research group I was part of was closed. In 1989, I started attending the European Carabidologists Meetings (ECM), this one hosted by Nigel Stork in London where, if I remember correctly, the delegation from over the ocean consisted only of John Spence. A bit later in 1995, probably because the meeting was billed as “The 3^rd^ International Symposium of Carabidology”, Terry and George were also present at the meeting organized by Jari Niemelä in Helsinki, Finland. Although this meeting did not land on the official list of numbered European Carabidologist Meetings, from then on, a small but strong delegation from North America continued to visit the European meetings.

I was very pleased these North American carabidologists turned out to be even more pleasant company than I expected. Where, according to my recollection, the Helsinki meeting in the evening was still divided along cultural lines into groups of Beer (Central and Northern Europe), Wine (Mediterranean Europe) and Spirits (America and some diehards, mostly East-Europeans) consuming people, these evening parties quickly became more mixed over the next meetings. Transatlantic cooperation and friendships grew rapidly. Terry and Grace were often there at the meetings, until the ECM in the Dolomites in Italy, organized by Roberto Pizzolotto and Pietro Brandmayr in 2019. Meanwhile the North Americans have been very well represented at the ECMs. We especially remember a post meeting excursion in Bulgaria in 2007, where we had the Erwin’s as guests in our car, visiting the fantastic places to which our host Lyubomir Penev directed us. A highlight, literally and figuratively, was the 1000-year-old Rila Monastir at altitude of 1100 meters, the most beautiful in the Balkans and probably of all southern Europe. Apparently taught by Grace, Terry showed himself also to be a rather good ornithologist, and it became clear that he knew the names of various European bird species that we saw during walks in the Rhodope mountains. We were impressed!

The importance of Terry’s scientific contributions is well known and these will be undoubtedly highlighted adequately in this memorial volume. Nevertheless, as a stunning example of Terry’s dedication to his favored taxon, I would like to mention his, unfortunately unfinished, series about the Caraboidea of the Western Hemisphere, the first parts of which were published by Pensoft. The set-up of this series could have come straight from Carl Lindroth, practical, extremely informative, beautifully illustrated, and not a word too much. This style was also very typical of Terry. Hopefully we will have something like this for Europe, one day.

In warm memory,

Hans and Annelies Turin

***Hans Turin*** *is a retired cardiologist who mainly works on the distribution and ecology of the European ground beetles, in particular on the fauna of the Netherlands and the surrounding area. His special sport is database work.*

**Figure:**
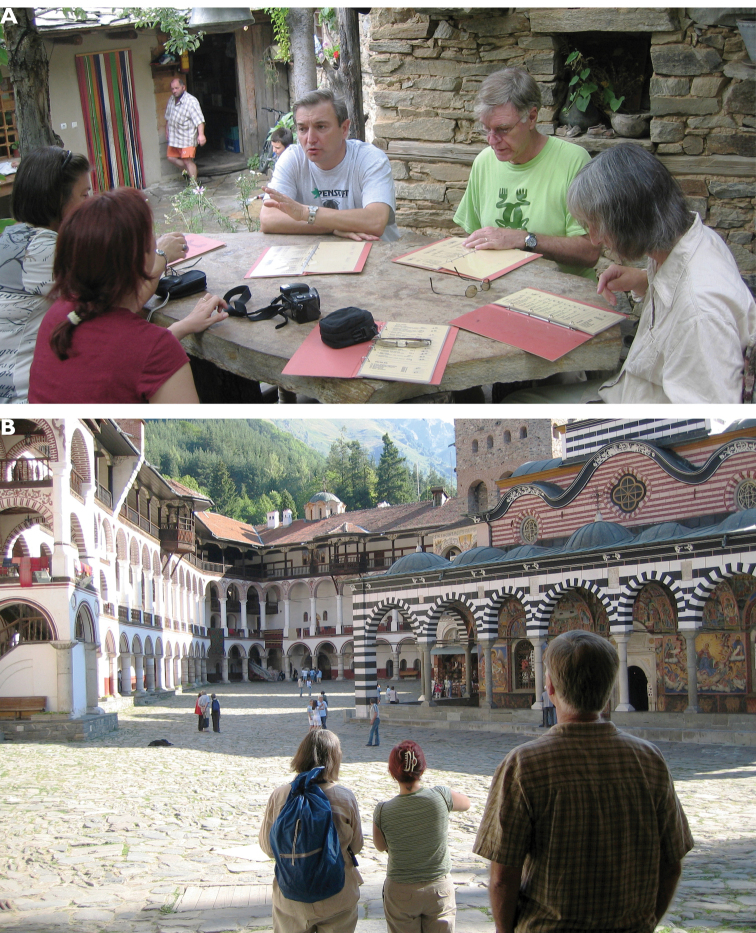
**Photograph captions.** Memories from the post-conference excursion after the XIII^th^ European Carabidologists meeting in Blagoevgrad, Bulgaria. August 2007 **A** Lunch in the village of Dolen, Bulgaria where *ZooKeys* was born (L to R, V. Peneva, G. Servat, L. Penev, T. Erwin, A. Turin) **B** Arrival at the 10^th^ C Eastern Orthodox Rila Monastery, a World Heritage Site (L to R, A. Turin, G. Servat, T. Ewin). Photographs: H. Turin.

## Helping me find a purpose for my life

I first heard about Terry Erwin when I was a first-year undergrad at college. Terry’s bioprospection of the Amazon canopy using fogging was introduced in lecture to illustrate the huge biodiversity in the tropics and the vast lacuna in our knowledge about insect diversity. As a young biology student that grew up in Colombia’s rainforest, the idea that the tropics could harbor up to 30 million of species of insects did not seem at all crazy to me! I was fascinated by this explosive diversity and puzzled by the evolutionary mechanisms that could lead to such incredible species richness. I decided then and there that I was going to find a way to participate in his research.

In 2011, I found my way to a summer internship at the Smithsonian National Museum of Natural History to work with Terry. During that summer, Terry introduced me to his ongoing research on the canopy entomofauna and assigned me the project of revising the cassidine beetles from the Ecuadorian canopy. That summer project resulted in the description of two new species and laid the grounds for a grant that allowed me to return to the Smithsonian the next year. Little did I know that internship was going to mark the beginning of what became a life-long mentorship, a long-term collaboration and close friendship.

Over the following years, Terry and I worked closely on an inventory of carabids beetles from the Ecuadorian Yasuní National park. During the many field trips to Tiputini Biological station in the heart of Yasuní, Terry not only shared with me his expansive knowledge about carabid taxonomy and biology but he also taught me to pay close attention to even the slight difference in the behavior of similar species, characters he used to accurately distinguish morpho-species by eye in the field!

I began to realize that in order to discern patterns in ecology and evolution one must begin with individual life histories; that general principles can only be inferred by the association of multifaceted data about how and where distinct creatures live. Terry taught that systematics is the basis for all meaningful studies of biodiversity, and that museum collections are a cradle for significant biological discoveries. Terry’s devotion to study of carabid diversity and his extensive work on carabid taxonomy constitute the pillars that could lead us to a deeper understanding of insect diversity in the tropics.

As it is often the case with mentors, their lessons transcend purely academic matters. To Terry I owe the love for research. From him I learned to be constantly fascinated by biological diversity, to try to commit to every single project, however small, as an end in itself, to focus on the task at hand with rigor and simplicity, and finally to pay attention to details. His example challenged me to find an equilibrium between effort and leisure. Terry helped amplify my love for the Amazon through feeling the joy that comes from being surrounded by living organisms, and through his influence I have come to know many people within the carabid academic family, many of whom have become lifetime friends.

The nine years that I was privileged to share with Terry shaped my career and life. I am committed to pursue the many projects we left unfinished in the coming years.

***Laura S. Zamorano****is currently a PhD student at CNRS, France. She was a student and a friend of Terry Erwin*.

**Figure:**
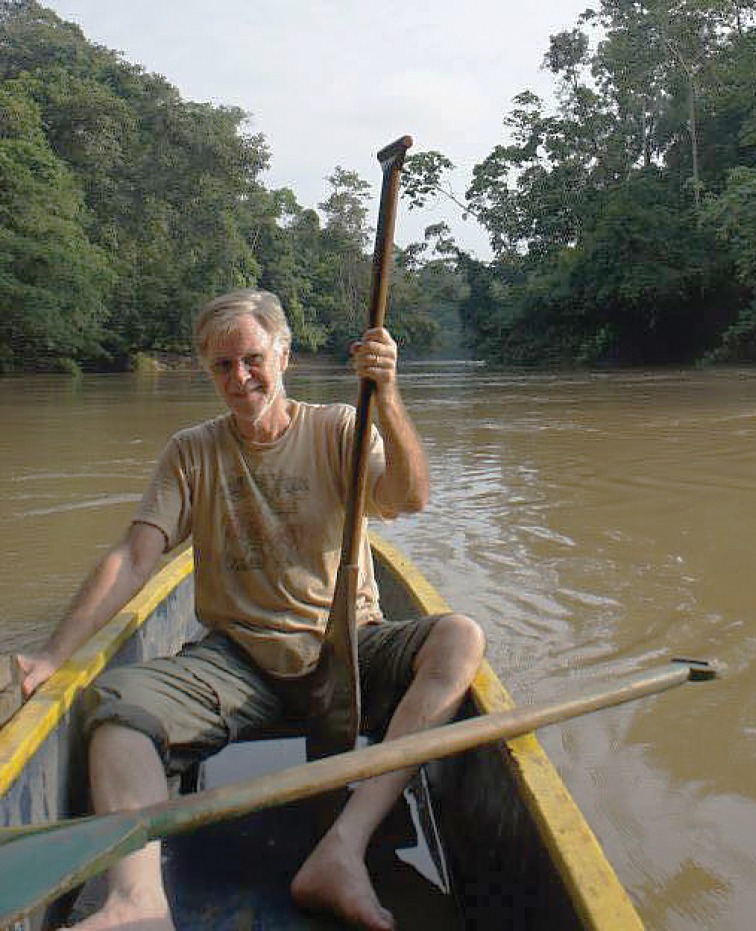
**Photograph caption.** Terry Erwin paddling down the Tiputini River in Yasuní, Ecuador. Terry and Laura Zamorano were on their way to sample carabid beetles in the igapó (black water system). Photograph: L. Zamorano.

